# Sacubitril/valsartan fails to prevent doxorubicin-induced cognitive impairment and hippocampal oxidative, inflammatory, and apoptotic alterations in rats

**DOI:** 10.3389/fphar.2025.1732842

**Published:** 2026-01-06

**Authors:** Mohrah Muteb Alresheedi, Maha Abdulrahman Aldubayan

**Affiliations:** Department of Pharmacology and Toxicology, College of Pharmacy, Qassim University, Buraydah, Saudi Arabia

**Keywords:** cognitive impairment, doxorubicin, neuroinflammation, oxidative stress, pro-apoptotic markers, sacubitril/valsartan

## Abstract

**Introduction:**

Doxorubicin (DOX) is a chemotherapeutic agent known for its effectiveness in treating various cancers. However, its clinical applicability is constrained by its neurotoxicity. DOX-induced toxicity is primarily driven by oxidative stress, inflammation, and mitochondrial dysfunction, resulting in elevated ROS and MDA, increased pro-inflammatory cytokines such as IL-6, IL-1β, TNF-α, and NF-κB, and increased apoptotic activity, including caspase-3 and BAX. Sacubitril/Valsartan (VS), a dual neprilysin inhibitor and angiotensin receptor blocker used in heart failure management, has shown protective effects by ameliorating inflammation and oxidative stress. This study aimed to investigate the potential of VS to mitigate or prevent hippocampal damage in rats.

**Methods:**

Forty male Wistar rats were randomly categorized into four groups (n = 10 per group): Control (normal saline), DOX (2.5 mg/kg), VS (60 mg/kg), and DOX + VS. VS was administered orally once daily via oral gavage, whereas DOX was delivered intraperitoneally weekly for 4 weeks. Body weight and survival were monitored daily. Cognitive performance was assessed using behavioral tests, followed by biochemical and histological analyses. Thereafter, oxidative, inflammatory, and pro-apoptotic markers were quantified.

**Result:**

DOX and VS co-administration resulted in significant reductions in body weight and survival compared with VS-treatment alone and controls. Furthermore, both DOX treatment alone and its co-administration with VS significantly increased hippocampal levels of oxidative, inflammatory, and apoptotic markers compared with VS treatment alone and controls. In addition, histopathological analysis revealed that hippocampal tissues subjected to DOX + VS treatment exhibited severe damage, comparable to that observed in tissues treated with DOX alone. In contrast, tissues treated with VS alone and controls showed less severe damage.

**Conclusion:**

Combining VS with DOX did not significantly enhance spatial learning and working memory compared with DOX alone, nor did it mitigate neuroinjury. These findings suggest that VS is not a viable therapeutic agent for alleviating DOX-induced neurotoxicity and cognitive dysfunction. This research offers novel insight for the field of pharmacological discovery by demonstrating that the neuroprotective potential of neprilysin inhibition (a key mechanism of VS) differs significantly from its cardioprotective actions. Our findings provide a foundational basis for the design of future neuroprotective therapies and for further research.

## Graphical Abstract

**Figure:**
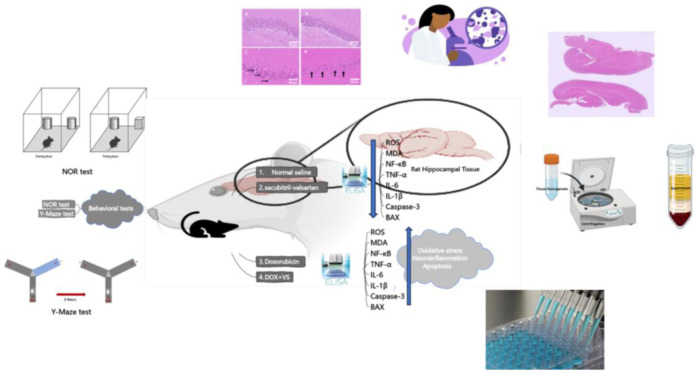

## 1 Introduction

Chemotherapy-induced cognitive impairment (“chemobrain”) is a well-recognized complication of cancer treatment and can persist long after therapy completion (Alsikhan et al., 2023). Doxorubicin (DOX), also known as adriamycin, is a chemotherapeutic agent with a significant impact on memory impairment. It belongs to the anthracycline class of anticancer drugs (Alhowail and Aldubayan, 2023; van der Zanden et al., 2021). Dox is a disorder characterized by a spectrum of symptoms, including memory loss, difficulty concentrating, impaired cognitive function, and other minor cognitive issues during and after chemotherapy (Bhatt et al., 2025; Boykoff et al., 2009). However, recent studies indicate that DOX-induced neurotoxicity is not confined to this patient group but may represent a broader issue affecting individuals undergoing treatment for various cancer types (Dietrich et al., 2015). This neurotoxicity is likely a contributing factor to chemotherapy-induced cognitive impairment, a condition that is estimated to impact between 17% and 75% of patients with cancer receiving chemotherapy (Kamińska and Cudnoch-Jędrzejewska, 2023). Several critical factors warrant consideration, including oxidative stress, inflammation, DNA damage, dysregulation of apoptosis, neurotransmitter imbalances, glial cell interactions, impaired neurogenesis, and epigenetic modifications (Du et al., 2021). These elements collectively contribute to the progression of chemotherapy-associated cognitive impairment, commonly termed “chemobrain” (Du et al., 2021).

DOX used in the treatment of various cancer types—activates multiple intracellular mechanisms of cell death, including the production of reactive oxygen species, DNA adduct formation, and the topoisomerase II inhibition (Aldubayan, 2023; Sritharan and Sivalingam, 2021). Despite its potential, the clinical use of this compound is restricted by its cytotoxicity on non-target tissues (Zhou et al., 2018). DOX exhibits poor blood‒brain barrier (BBB) permeability, which limits its brain penetration and results in toxicity primarily associated with peripheral effects (K. Kamińska & A. Cudnoch-Jędrzejewska, 2023). The resultant effects include cognitive deficits and structural modifications in the nervous system, as supported by evidence from both preclinical and clinical research (Kamińska and Cudnoch-Jędrzejewska, 2023). DOX-associated neurotoxicity can directly impact neurons through interactions with the cell body or indirectly via processes such as glial damage and inflammation (Alsaud et al., 2023; Was et al., 2022). These effects may lead to a spectrum of symptoms that negatively impact the quality of life for individuals undergoing treatment (Kamińska and Cudnoch-Jędrzejewska, 2023). Although various forms of neurotoxicity, including those affecting the enteric nervous system or the brain, have historically been neglected, there has been a recent shift towards a more profound understanding of the fundamental mechanisms underlying neurotoxicity (Was et al., 2022).

DOX is known to induce significant oxidative stress in the hippocampus, primarily by elevating reactive oxygen species (ROS) levels. During the redox cycling of DOX, it generates elevated levels of ROS, such as superoxide and hydrogen peroxide, which surpass the neuronal antioxidant capacity and disrupt cellular homeostasis (Sritharan and Sivalingam, 2025). The principal consequence of this oxidative overload is lipid peroxidation, as evidenced by increased levels of malondialdehyde (MDA), a widely used marker of oxidative membrane damage. The elevation of ROS and MDA in the hippocampus has been consistently documented in preclinical models of DOX-induced neurotoxicity. It is strongly correlated with neuronal structural damage and cognitive impairment (Alhowail and Aldubayan, 2023). This state of oxidative stress predisposes the system to subsequent inflammatory activation and neuronal apoptosis.

In addition to oxidative dysregulation, inflammatory TNF-α can influence hippocampal volume and interfere with long-term potentiation in both the CA1 region and the dentate gyrus (Du et al., 2021). The presence of circulating TNF-α facilitates its passage across the BBB via endothelial cells that express TNF-α receptors (Du et al., 2021). Upon entering the brain, TNF-α binds receptors on glial cells, thereby enhancing inflammatory signaling by activating astrocytes and microglia, leading to localized TNF-α production (Du et al., 2021). This interaction recruits intracellular proteins, thereby initiating inflammatory pathways that lead to the nuclear translocation of nuclear factor kappa β (NF-κβ) (Du et al., 2021). Furthermore, brain inflammation may suppress the expression of inducible nitric oxide synthase through the transcriptional NF-κβ activity, leading to oxidative and nitrative stress within the brain (Du et al., 2021). The resultant oxidative stress triggers NF-κB activation, which stimulates the secretion of pro-inflammatory cytokines, including TNF-α, IL-1β, and IL-6 (Du et al., 2021; Liu et al., 2017). Experimental investigations have demonstrated that DOX administration in mice results in elevated plasma TNF-α levels within 1 h post-treatment (Tangpong et al., 2006). NF-κB is often associated with inflammatory processes implicated in various neuroinflammatory disorders (Li et al., 2022). Notably, research indicates that inhibiting NF-κB can decrease neuroinflammatory markers and improve cognitive performance in models of DOX-induced cognitive impairment (McAlpin et al., 2022).

Subsequently, these oxidative and inflammatory insults converge on intrinsic apoptotic pathways, leading to increased caspase-3 activity and the upregulation of pro-apoptotic markers such as BAX, which contribute to hippocampal neuronal degeneration and cognitive decline (Alhowail and Aldubayan, 2023; Radeva and Yoncheva, 2025). Current clinical interventions aimed at addressing DOX-induced neurotoxicity have demonstrated only limited effectiveness (El-Agamy et al., 2019). Therefore, it is essential to develop therapeutic strategies that effectively alleviate DOX-associated neurotoxic effects and cognitive impairments.

Sacubitril/valsartan (VS) (LCZ696) represents an innovative therapeutic option for heart failure, although its precise mechanisms remain unknown (Nielsen et al., 2018). VS operates by enhancing the natriuretic peptide system through neprilysin inhibition, while simultaneously mitigating the renin-angiotensin-aldosterone system by blocking the angiotensin II receptor (Galo et al., 2021; Iborra-Egea et al., 2017; Singh et al., 2017). VS is rapidly converted into its active metabolite LBQ657, which has been shown to cross the BBB to a limited extent. Detectable LBQ657 concentrations were found in the cerebrospinal fluid of both animals and humans, suggesting central neprilysin inhibition and potential neuroinflammation pathway modulation (Poorgolizadeh et al., 2021). VS primarily influences cellular activities by modulating the NF-κB signaling pathway. NF-κB a crucial transcription factor, plays a significant role in regulating the expression of various pro-inflammatory cytokines, including TNF-α, IL-1β, and IL-6, which are pivotal in neuroinflammatory processes (Bai et al., 2021). Furthermore, the cardioprotective effects of VS encompass oxidative stress reduction, which is frequently intensified by inflammatory processes (Yu et al., 2020). The combination of neprilysin inhibition and angiotensin receptor blockade may enhance nitric oxide bioavailability, thereby improving vascular function and reducing oxidative damage in cardiac tissues (Trivedi et al., 2018). Prior research has demonstrated that LCZ696 may impede the pathological progression of hepatic fibrosis by attenuating oxidative stress, inflammation, and hepatic NF-κB activation (Alqahtani et al., 2020). Moreover, recent studies have investigated the cardioprotective effects of VS, highlighting mechanisms such as mitochondrial quality enhancement, oxidative stress reduction, autophagy and mitophagy pathway regulation, and modulation of the natriuretic peptide system (Tantisuwat et al., 2023).

**FIGURE 1:**
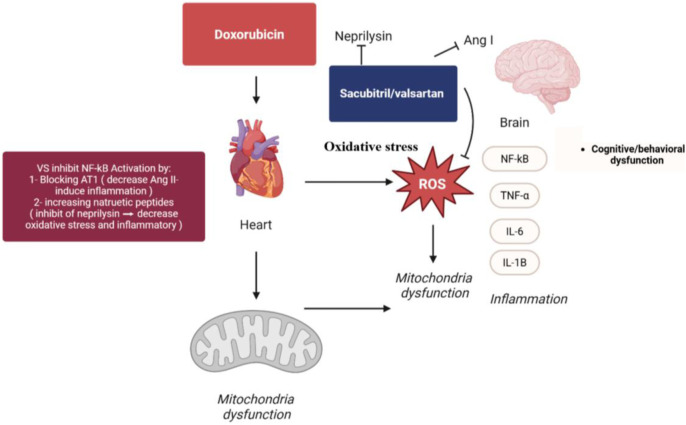
Proposed neuroprotective effects of VS in DOX-induced toxicity.

Although VS exhibits limited direct penetration across the BBB, its potential CNS benefits may arise through indirect mechanisms. VS modulates systemic oxidative and inflammatory pathways via dual inhibition of the renin–angiotensin system and neprilysin, leading to reduced peripheral cytokine release and vascular oxidative stress that secondarily influences neuroinflammation (Shi et al., 2024). Moreover, preclinical studies have demonstrated that VS improves cognitive performance and attenuates neuronal injury in models of Alzheimer’s disease and ischemic brain injury despite low BBB permeability (Zhang et al., 2023). Therefore, the rationale for using VS in CNS injury models lies in its ability to modulate peripheral neuroinflammatory and vascular pathways that indirectly confer neuroprotection and enhance mitochondrial function, implicated in cognitive impairment and neurodegenerative disorders (Figure 1).

VS reportedly modulates oxidative stress, inflammatory and pro-apoptotic pathways in the heart, hepatic, and lung, enhancing mitochondrial function and reducing oxidative stress. Despite these findings, the potential of VS to mitigate DOX-induced chemobrain remains unknown. Therefore, this study aimed to investigate the effects of VS and elucidate the mechanisms underlying their influence on DOX-induced cognitive deficits.

## 2 Materials and methods

### 2.1 Drugs and chemicals

DOX hydrochloride injection (50 mg/25 mL; 2 mg/mL) was obtained from Ebewe Pharma Ges.m.b.H. Nfg. KG, Unterach am Attersee, Austria, and VS (Entresto®, 100 mg film-coated tablets) were purchased from Novartis Pharma AG, Basel, Switzerland.

### 2.2 Animals

This study involved 40 male Wistar rats, each weighing 200–380 g and aged 10–12 weeks, sourced from the College of Pharmacy Animal House at Qassim University, Saudi Arabia. The rats were housed in propylene cages (four rats per cage) and maintained at 25 °C ± 2 °C under a 12-h light-dark cycle. They were given unrestricted access to food and water. Daily monitoring was conducted to track body weight and mortality rates, and behavioral assessments were conducted during the light cycle. The experimental procedures were approved by the Deanship of Graduate Studies and Scientific Research, Qassim University (Approval No. 25-37-03) and conducted in accordance with the National Institute of Health guidelines for the Care and Use of Laboratory Animals.

### 2.3 Experimental groups and treatment schedule

The animals were randomly assigned to four groups (n = 10 each): Control, DOX, VS, and DOX + VS. The control animals received an equivalent volume of normal saline by oral gavage once daily for 4 weeks. The DOX group was treated with DOX (2.5 mg/kg/week intraperitoneally) for 4 weeks (cumulative dose, 10 mg/kg) every week for four cycles (Moretti et al., 2021). The VS group (Entresto, 100 mg per tablet) was freshly prepared each day before dosing. Tablets were finely powdered and suspended in 10 mL of distilled water to yield a final concentration of 100 mg/10 mL. The required dose was calculated for each rat based on its body weight. Animals received 60 mg/kg/day by oral gavage for 28 days (Burke et al., 2019). The combination group received DOX (2.5 mg/kg) by intraperitoneal injection, 4 cycles every 7 days, and VS (60 mg/kg) orally once daily for 28 days. The animals were subjected to behavioral evaluations of cognitive function using the Y-maze and the novel object recognition (NOR) test.

### 2.4 Mortality rate and body weight

Continuous monitoring of mortality rates was crucial for the study’s progression. Cage maintenance was performed twice daily to ensure the prompt removal of any deceased animals. Forty male Wistar rats (200–380 g) had their body weight measured daily and were systematically recorded to assess overall health, enabling early detection of subtle changes and prompt identification of potential health issues.

### 2.5 Behavioral assessment

Spatial working memory and recognition memory were evaluated using the Y-maze and Novel Object Recognition (NOR) tests. All behavioral sessions were recorded using an HP 320 FHD Webcam (Full HD 1080p, 66° wide-angle lens; HP Inc., United States), and behavioral parameters were analyzed using EthoVision XT (Version 16, Noldus Information Technology, Netherlands). Statistical analyses and graph generation were performed using GraphPad Prism (Version 10, GraphPad Software, United States).

#### 2.5.1 Y-mazeTest

**FIGURE 2:**
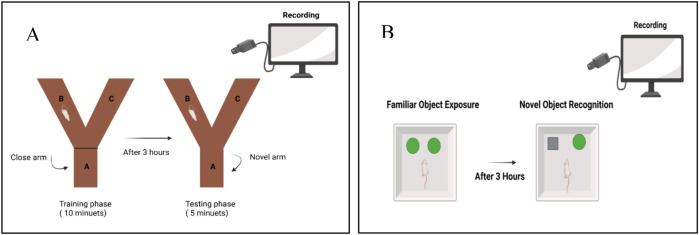
Schematic representation of the behavioral assessment procedures used in this study. **(A)** Y-maze spontaneous alternation test. **(B)** Novel Object Recognition (NOR) test.

Spatial and working memory were assessed through spontaneous alternation behavior in a Y-shaped wooden maze (50 × 10 × 18 cm) with three arms arranged at 120°. During the training phase, each group of rats (n = 7) was allowed to explore two open arms for 10 min while the third arm remained closed. After a 3-h interval, a 5-min test session was conducted in which all three arms were accessible, designating the previously closed arm as the *novel arm*. The number of entries into the novel arm, the time spent in the novel arm, and the total number of arm entries were recorded and analyzed (Alotayk et al., 2023) (Figure 2A).

#### 2.5.2 NOR test

Recognition memory was evaluated in an open wooden box (40 × 40 × 40 cm) containing two distinct objects. During the familiarization phase, animals freely explored two identical objects for 10 min. Following a 3-h retention interval, a 5-min test session was performed in which one of the familiar objects was replaced with a novel object. Time spent exploring the novel versus the familiar object was quantified for each rat (n = 7). To eliminate olfactory cues, the arena and objects were cleaned with 70% ethanol between trials (Figure 2B).

### 2.6 Hippocampus tissue extraction and collection for biochemical analysis

Upon completion of the experiment, the rats were euthanized through CO_2_ asphyxiation in a glass chamber (Conlee et al., 2005). Thereafter, the brains were carefully removed and washed with ice-cold phosphate-buffered saline to clear any remaining blood. The hippocampal tissue was extracted and subsequently homogenized using a Qsonica homogenizer (30 Hz, Newtown, CT, United States) with N-PER lysis buffer (Thermo Scientific, Madison, WI, United States). Following homogenization, the mixture was centrifuged at 12,000 × *g* for 10 min, and the supernatant was collected and stored at −80 °C.

### 2.7 Histopathology evaluation and scoring of hippocampus injury

Hippocampal (n = 7) samples were promptly fixed in 10% neutral buffered formalin for 24–48 h post-fixation, the tissues underwent dehydration through a series of increasing ethanol concentrations, were cleared in xylene, and subsequently embedded in paraffin wax. Serial coronal sections, with a thickness of 4–5 μm, were prepared using a rotary microtome. These sections were mounted on glass slides, deparaffinized, rehydrated, and stained with hematoxylin and eosin (H&E) according to standard histological procedures. The degree of hippocampal injury was evaluated using the Suzuki scoring system (Toledo-Pereyra and Suzuki, 1994). The scale ranges from 0 (none) to 4 (severe). All slides were examined blindly, and the mean score was used for analysis. The stained slides were examined under a light microscope at ×400 magnification, and representative images were captured.

### 2.8 CA1 neuronal density analysis

Neuronal density within the hippocampal CA1 region was quantified utilizing ImageJ software (NIH, United States). Images were captured at ×400 magnification, and each image was calibrated using a 50 µm scale bar prior to analysis. A standardized rectangular region of interest (ROI; 1.52 mm^2^) was positioned over the CA1 pyramidal cell layer to ensure consistent ROI dimensions across all groups. Neurons were manually counted using the ImageJ Cell Counter plugin, with inclusion criteria limited to neurons exhibiting a clearly identifiable soma and nucleus. Glial cells, pyknotic bodies, and fragmented debris were excluded from the count. Neuronal density (cells/mm^2^) was determined by dividing the number of neurons within the ROI by the ROI area. For each animal, seven non-overlapping ROIs were analyzed, and mean values were employed for statistical comparison.

### 2.9 Enzyme-linked immunosorbent assay (ELISA)

The sandwich ELISA method was applied as a precise biochemical technique to assess the concentration of oxidative stress, inflammatory, and apoptotic mediators. This study quantified the levels of ROS, MDA, IL-1β, IL-6, TNF-α, NF-κB, caspase-3, and BAX in hippocampal tissue homogenates (n = 7) from each group using rat-specific ELISA kits commercially available, analyzed for oxidative stress biomarkers, including MDA [Cat no. RK15281], ROS [Cat no. RK15283], (IL-1β [cat.no. ELK1272], IL-6 [cat.no. ELK1158], TNF-α [cat.no. ELK1396], and NF-κB [cat.no. ELK1693], caspase-3 [Cat. No. RK03549], and BAX [Cat. No. RK03522]; ELK Biotechnology, Denver, CO, United States). Absorbance was determined at 450 nm using a microplate reader (BioTek Instruments, United States) (Aldubayan, 2025b; Alhowail et al., 2025). All experimental procedures were conducted strictly according to the manufacturer’s instructions.

### 2.10 Statistical analysis

All results are presented as the mean ± standard error of mean and were analyzed utilizing GraphPad Prism 10 software (GraphPad, Boston, MA, United States). The data, encompassing body weights, survival rates, Y-maze and NOR behavioral tests, and biochemical assays, were subjected to one-way analysis of variance (ANOVA) followed by the Tukey–Kramer test for multiple comparisons. A p-value <0.05 was considered indicative of statistical significance.

## 3 Result

### 3.1 Effect of DOX and VS on body weight and mortality

**FIGURE 3:**
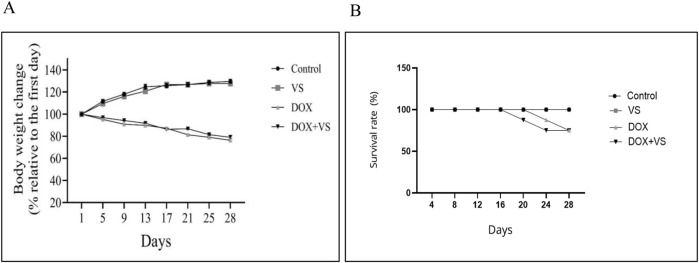
**(A)** Effect of VS and DOX on rat body weight. **(B)** Effect of VS and DOX on rat survival rate.

Compared with the control group, both the DOX and DOX + VS groups demonstrated a reduction in body weight, whereas the VS group exhibited an increase in body weight (Figure 3A). Furthermore, elevated mortality rates were observed in the DOX and DOX + VS groups daily (Figure 3B). Specifically, the DOX group had a 70% survival rate, while both the VS and control groups maintained 100% survival. The DOX and DOX + VS groups showed increased mortality rates after the third DOX dose compared with the VS-treated alone and the control group.

### 3.2 Effect of DOX and VS on Y-maze performance

**FIGURE 4:**
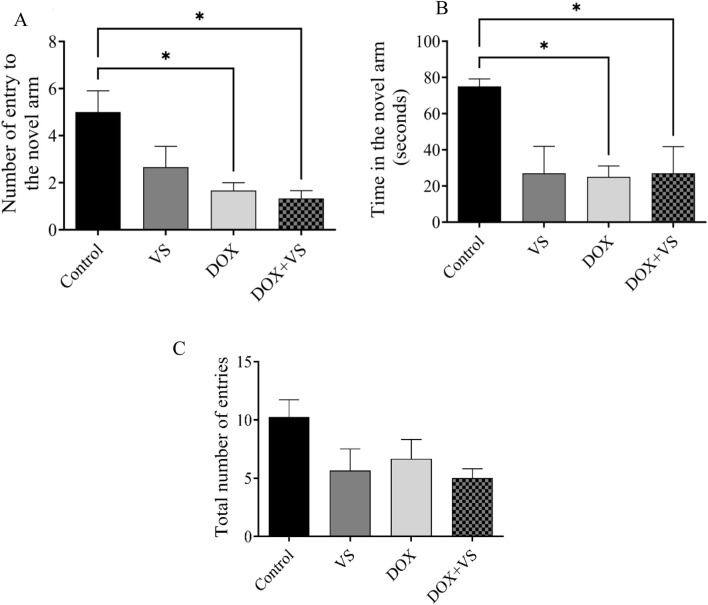
The impact of VS on DOX-induced cognitive and behavioral impairment was assessed in rats using the Y-maze task. **(A)** Effect of VS and DOX on the total number of entries to the novel arm. **(B)** Effect of VS and DOX on the time spent in the novel arm. **(C)** Total entries across all arms. Data are expressed as the mean ± SEM. Statistical analysis was conducted using a one-way ANOVA followed by a Tukey–Kramer post-hoc test, with differences deemed significant at p < 0.05 compared with the control group (n = 7).

In the DOX and DOX + VS treatment groups, a significant reduction (p < 0.05) was observed in both the duration and frequency of entries into the novel arm when compared with the control group (Figures 4A,B). No significant differences were detected among the VS, DOX, and DOX + VS groups. In addition, the total number of entries into all arms did not significantly vary across all groups (Figure 4C), indicating that the reductions in novel arm exploration were not due to a decrease in overall exploratory behavior.

### 3.3 Effect of DOX and VS on NOR test performance

**FIGURE 5:**
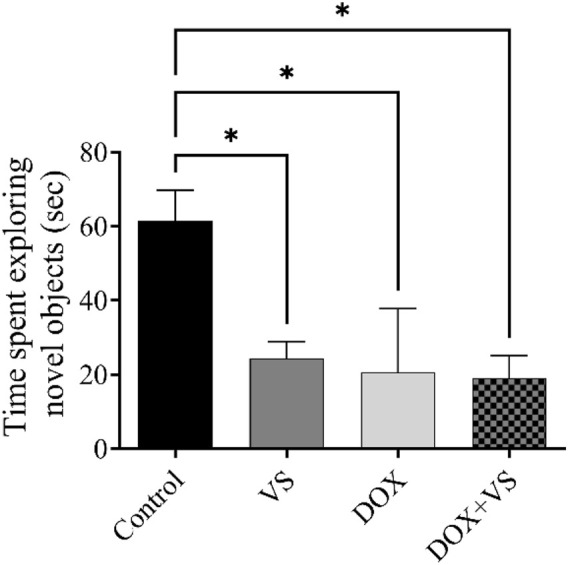
Impact of VS administration on DOX-induced changes in cognitive performance in rats, as assessed by the NOR test. Data are expressed as the mean ± SEM (n = 7). Statistical analysis was conducted using one-way ANOVA followed by the Tukey–Kramer *post hoc* test. Significant differences were observed at *p < 0.05 when compared with the control group (n = 7).

In the NOR test (Figure 5). The control group demonstrated significantly greater exploration time of the novel object than the VS, DOX, and DOX + VS groups (p < 0.05).

### 3.4 Histopathology

**FIGURE 6:**
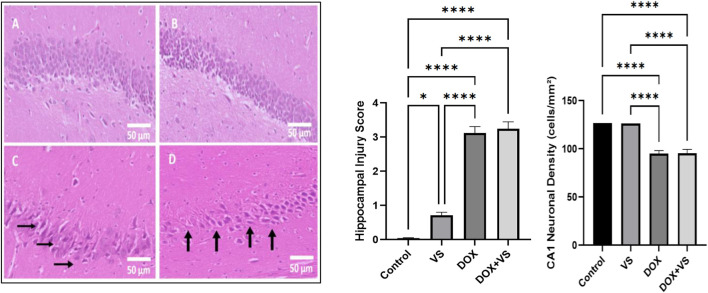
Representative photomicrographs of CA1 hippocampal sections stained with hematoxylin and eosin (scale bar = 50 µm) **(A)** Control group exhibiting normal histoarchitecture of the pyramidal cell layer with intact neuronal morphology. **(B)** The VS-treated group showed preserved neuronal density and architecture with only minimal alterations. **(C)** DOX-treated group demonstrating marked neuronal injury (arrows), characterized by shrunken eosinophilic neurons, nuclear pyknosis, vacuolated neuropil, and disorganization of the pyramidal cell layer. **(D)** DOX + VS-treated group showing persistent neuronal degeneration and loss of pyramidal cell organization (arrows), with no significant improvement compared with the DOX group. Data are expressed as the mean ± SEM (n = 7) and analyzed using one-way ANOVA followed by Tukey’s *post hoc* test, with statistical significance set at ****p < 0.0001, and *p < 0.05 compared with the control group.

Histopathological assessment of the hippocampal CA1 region revealed marked structural alterations following DOX treatment. The control group exhibited a well-organized, densely packed pyramidal cell layer with intact neuronal morphology, whereas the VS-treated group showed comparable cytoarchitecture, indicating no adverse neuronal effects. In contrast, DOX-treated rats showed pronounced neurodegenerative changes, including shrunken eosinophilic neurons, nuclear pyknosis, neuropil vacuolation, and severe disorganization of the pyramidal cell layer. The DOX + VS group demonstrated persistent neuronal degeneration and disrupted pyramidal alignment, with no observable improvement relative to the DOX group. These qualitative findings align closely with quantitative measures of neuronal density, which showed a significant reduction in CA1 neurons in the DOX group, with no meaningful restoration in the DOX + VS group. Quantitative analysis of CA1 neuronal density (cells/mm^2^) revealed a significant reduction in DOX-treated rats compared with controls, whereas VS alone had no effect. Co-treatment with VS failed to restore neuronal density in DOX-exposed rats (Figure 6).

### 3.5 Impact of VS in conjunction with DOX on oxidative stress markers in rat hippocampal tissues

**FIGURE 7:**
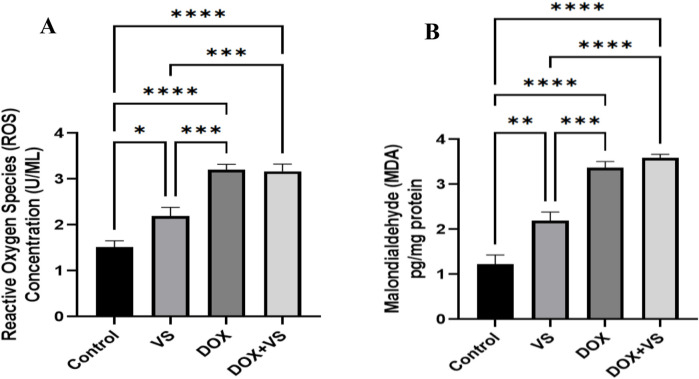
Impact of VS administration on DOX-induced alterations in oxidative stress markers. **(A)** ROS, and **(B)** MDA in rat brain tissue. Data are expressed as the mean ± SEM (n = 7) and analyzed using one-way ANOVA followed by Tukey’s *post hoc* test, with statistical significance set at ****p < 0.0001, ***p < 0.001, and *p < 0.05 compared with the control group.

In DOX-treated animals, a significant elevation was observed in ROS and MDA levels compared with those treated with VS alone and controls. Furthermore, the DOX + VS-treated groups showed no significant difference in ROS and MDA levels when compared with the DOX group. Conversely, a substantial increase was observed compared with the VS and control groups (Figures 7A,B).

### 3.6 Impact of VS in conjunction with DOX on inflammatory markers in rat hippocampal tissues

**FIGURE 8:**
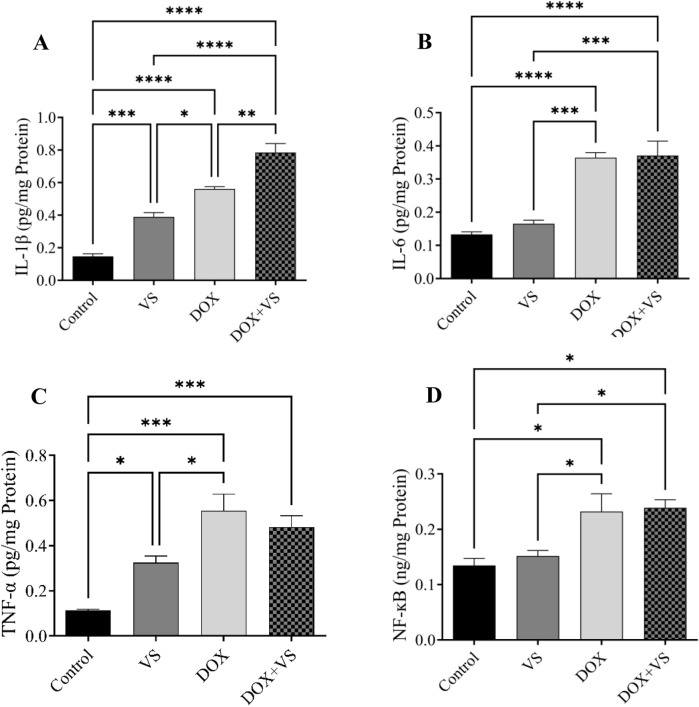
Impact of VS administration on DOX-induced alterations in neuroinflammatory markers. **(A)** IL-1β, **(B)** IL-6, **(C)** TNF-α, and **(D)** NF-κB in rat brain tissue. Data are expressed as the mean ± SEM (n = 7) and analyzed using one-way ANOVA followed by Tukey’s *post hoc* test, with statistical significance set at ****p < 0.0001, ***p < 0.001, **p < 0.01, and *p < 0.05 compared with the control group.

In DOX-treated animals, a significant elevation was observed in IL-1β, IL-6, TNF-α, and NF-κB levels compared with those treated with VS alone and controls. However, DOX + VS administration resulted in a significant increase in IL-1β levels compared with all other groups. Furthermore, no significant differences were observed in IL-6, TNF-α, or NF-κB levels compared with the DOX group. Conversely, a substantial increase was observed compared with the VS and control groups (Figures 8A–D).

### 3.7 Impact of VS in conjunction with DOX on apoptotic markers in rat hippocampal tissues

**FIGURE 9:**
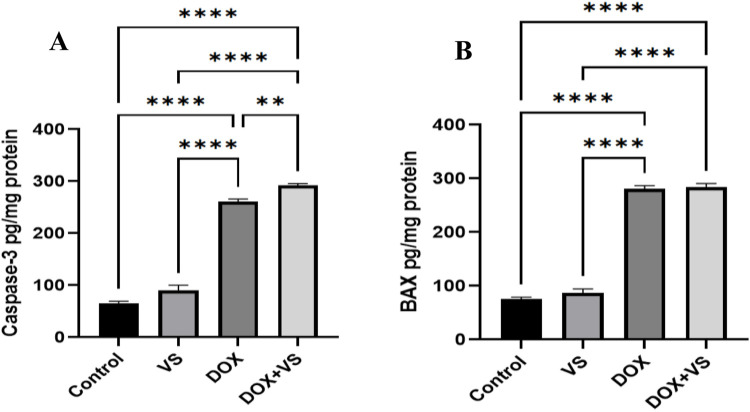
Impact of VS administration on DOX-induced alterations in apoptotic markers. **(A)** caspase-3 and **(B)** BAX in rat brain tissue. Data are expressed as the mean ± SEM (n = 7) and analyzed using one-way ANOVA followed by Tukey’s *post hoc* test, with statistical significance set at ****p < 0.0001 and **p < 0.01 compared with the control group.

In DOX-treated animals, a significant elevation was observed in caspase-3 and BAX compared with those treated with VS alone and controls. However, DOX + VS administration resulted in a significant increase in caspase-3 compared with all other groups. Further, no significant difference was observed in BAX when compared with the DOX group (Figures 9A,B).

## 4 Discussion

This study evaluated the potential neuroprotective effect of VS against DOX-induced cognitive impairment in rats, focusing on oxidative stress, pro-inflammatory cytokines, and apoptosis. Behavioral, biochemical, and histopathological findings consistently demonstrated that VS failed to ameliorate DOX-induced neurotoxicity or cognitive deficits. DOX-induced “chemobrain” is associated with a significant change in oxidative stress, inflammatory, and apoptotic markers in the brain, such as elevated ROS, MDA, IL-1β, IL-6, TNF-α, NF-κB, caspase-3, and BAX levels (Alhowail and Aldubayan, 2023; Radeva and Yoncheva, 2025). The use of DOX in rats reportedly results in chemotherapy-induced brain impairment, which closely resembles the clinical manifestations observed in patients receiving DOX treatment (Kesler et al., 2013). This animal model provides a valuable preclinical framework for investigating the potential effects of VS in a clinically relevant context (Kesler et al., 2013). However, our findings indicate that VS did not exert any protective influence on DOX-induced behavioral impairment or the oxidative stress, neuroinflammatory, and apoptotic pathways.

To date, this study is the first to investigate the potential effect of VS on DOX-induced cognitive impairment. Previous research exploring the impact of VS has mainly focused on its cardio- and hepatic-protective effects; for example, they found that VS can attenuate myocardial inflammation, fibrosis, and apoptosis, and promote autophagy in DOX-induced cardiotoxicity (Hu et al., 2025). Moreover, other studies have demonstrated that VS administration in rat models provides hepatic protection by reducing oxidative stress, suppressing inflammation, and NF-κB activation (Alqahtani et al., 2020). Only a few studies have examined the potential association between VS and cognitive impairment. For instance, the effect of VS on cognitive impairment in the colchicine-induced Alzheimer model in rats (Hammadi et al., 2023). These findings are consistent with our results, which demonstrated the cognitive deficits with VS treatment. Clinical evidence regarding VS and cognition remains inclusive. A recent nationwide cohort study by Jung et al. (2025) reported a reduced risk of dementia in patients with heart failure treated with VS, suggesting potential long-term cognitive benefits in humans (Jung et al., 2025). In contrast, the PARADIGM-HF cognitive found no significant difference between the VS and comparator groups (Garnier-Crussard, 2024). These clinical findings support the notion that the central nervous system (CNS) effects of VS are complex and may depend on factors such as BBB permeability, patient age, comorbidities, and duration of exposure (Garnier-Crussard, 2024; Jung et al., 2025).

DOX administration resulted in a marked reduction in body weight throughout the experiment, likely due to anorexia, gastrointestinal toxicity, and systemic inflammation associated with its use. The survival rate was approximately 70% in both the DOX and DOX + VS groups, whereas all animals in the control and VS groups survived (100%). These findings indicate the systemic toxicity of DOX and confirm the validity of this model for studying chemotherapy-induced complications (Alsikhan et al., 2023; Demos-Davies et al., 2022; Hajjaji et al., 2012). Despite the increased hippocampal neuro-oxidative stress and neuroinflammation observed in the DOX + VS group, the mortality rate remained consistent with that of the DOX group alone (30%). This outcome is anticipated, as mortality in DOX-treated animals is predominantly attributed to systemic toxicity, including cardiotoxicity, nephrotoxicity, gastrointestinal injury, hematological suppression, and immune dysfunction, rather than central nervous system-specific injury (Hajjaji et al., 2012; Demos-Davies et al., 2022). The timing and pattern of mortality were similar between the DOX and DOX + VS groups, suggesting that VS did not exacerbate DOX’s systemic toxicity. Notably, there is no evidence indicating that VS alters the pharmacokinetics or systemic burden of DOX (McMurray et al., 2014; Han et al., 2016). Therefore, although VS worsens the oxidative and neuroinflammatory profile, it does not affect survival, which remains governed by DOX-induced peripheral organ toxicity.

To evaluate the impact of DOX and VS on cognitive function in rats, a series of hippocampal-dependent behavioral paradigms, including the Y-maze and NOR Test, were used as established proxies for assessing short-term and recognition memory (Alotayk et al., 2023). Given the known neurotoxic effect of DOX, alterations in these behavioral outcomes are indicative of hippocampal dysfunction and broader cognitive impairment. In the Y-maze task, DOX-treated rats showed a reduced number of entries into the novel arm compared with controls. Furthermore, the time spent in the novel arm differed significantly from that of the control group; these findings suggest that DOX administration induced cognitive impairment in rats, whereas the control group exhibited preserved cognitive performance. Moreover, the addition of VS to DOX did not modify these outcomes. To ensure that the observed deficit in number was not attributable to reduced locomotor activity, the total number of arm entries was analyzed, and no significant difference was observed among the groups.

In the NOR test for short memory function, rats in the DOX and DOX + VS groups spent less time exploring the novel object compared with controls. Although the treatment group did not improve the exploration time of the novel object, this test demonstrated that DOX + VS was unable to ameliorate cognitive impairment. The outcome of these behavioral tests confirmed DOX-induced cognitive impairment, consistent with previous studies (Alhowail and Aldubayan, 2023; Alhowail et al., 2021). In addition to the effects observed in the DOX-treated groups, the NOR test revealed that VS alone significantly impaired recognition memory compared to controls. This reduction in exploratory preference for the novel object suggests that VS may adversely affect hippocampal-dependent cognitive performance even in the absence of DOX. Mechanistically, this finding is consistent with our biochemical results, which demonstrated that VS monotherapy significantly increased hippocampal ROS, MDA, IL-1β, and TNF-α levels. These elevations likely reflect neprilysin-inhibition mediated accumulation of neuroactive peptides (Iwata et al., 2001; Nalivaeva et al., 2020; Skidgel and Erdös, 2004), resulting in low-grade oxidative stress and microglial activation that disrupts recognition memory. Furthermore, no significant differences were observed between the VS, DOX, and DOX + VS groups in the NOR test, suggesting that DOX-induced neurotoxicity may reach a ceiling effect, beyond which cognitive impairment cannot further deteriorate. Collectively, these findings suggest that both DOX-induced neuro-injury and VS-induced neuropeptide accumulation can independently compromise hippocampal function, leading to similar reductions in NOR performance across all treatment groups.

In our study, histopathology staining results for the DOX + VS-treated group showed severe damage, indicated by a black arrow, similar to that observed in tissues treated with DOX alone, as opposed to those treated with VS alone or controls. A similar finding was reported in a previous study on DOX, which examined the hippocampus and revealed evident structural impairment in the brain (Mohamed et al., 2011; Moretti et al., 2021), suggesting the ineffectiveness of VS in protecting the brain against DOX-induced impairment. Conversely, other studies have reported that the VS may play an essential role in improving cognitive impairment by downregulating the neuroplasticity-related pathways associated with diabetes in rats (Al-Ashram et al., 2025). Furthermore, the significant reduction in CA1 neuronal density following DOX administration aligns with existing evidence indicating that hippocampal pyramidal neurons are susceptible to oxidative stress, mitochondrial dysfunction, and apoptotic signaling induced by DOX. The maintained neuronal density in the VS group corroborates that VS does not exert neurotoxic effects on hippocampal neurons under physiological conditions. Notably, VS’s inability to restore CA1 neuronal architecture or density in DOX-treated rats suggests that neprilysin inhibition and AT1 receptor blockade do not protect hippocampal neurons against DOX-induced neurodegenerative pathways. This observation is consistent with the pronounced histopathological abnormalities observed in the DOX + VS group. It supports the conclusion that VS does not provide neuroprotection in the context of DOX-induced central neurotoxicity.

In the context of oxidative stress, the administration of DOX significantly increased the production of ROS and MDA levels within the hippocampus, thereby corroborating the established role of oxidative damage in DOX-induced neurotoxicity (Alhowail et al., 2019; Alhowail and Aldubayan, 2023), which aligns with our findings. Conversely, the concurrent administration of VS did not ameliorate or rectify these oxidative alterations, indicating its lack of efficacy in modulating DOX-induced oxidative stress pathways. The inability of VS to reduce DOX-induced ROS and MDA may be attributed to the insufficient penetration of its active metabolite (LBQ657) across the BBB, which impedes the modulation of central antioxidant pathways (Jung et al., 2025).

In addition to the oxidative stress profile, the elevation of inflammatory biomarkers such as IL-6, IL-1β, TNF-α, and NF-κB levels is associated with CNS injury and cognitive behavioral deficits (Aldubayan, 2025a; Alhowail et al., 2025; Babić Leko et al., 2020; Choi et al., 2015). In our study, these biomarkers were significantly increased in the DOX group compared with the control group, consistent with previous studies that reported an increase in neuroinflammatory markers (Alsikhan et al., 2023). The increase in TNF-α following DOX administration disrupted the BBB, facilitating its entry into brain tissue, particularly the hippocampus. Consequently, TNF-α production further promoted the production of other inflammatory mediators through NF-κB activation, leading to increased IL-1β and IL-6 levels. This cascade of neuroinflammation is strongly associated with the development of cognitive impairment (Alsikhan et al., 2023; Du et al., 2021).

In this study, the DOX + VS group had no significant effect on TNF-α levels compared with the DOX group alone. However, TNF-α levels were significantly higher than in the control group. This finding suggests that the drug did not confer protection by reducing TNF-α levels, in contrast to previous studies on the heart, which reported TNF-α reductions (Hu et al., 2025). The effect of sacubitril on reducing inflammatory markers in the heart may be due to its action on neprilysin, thereby modulating the levels of protective peptides. However, its limited ability to cross the BBB prevents it from exerting a similar anti-inflammatory effect in the brain, particularly in the hippocampus (Bunsawat et al., 2021; Jung et al., 2025). IL-6 and IL-1β levels were significantly increased in the DOX and DOX + VS-treated groups compared with controls, attributed to cognitive dysfunction in the brain, which led to their subsequent elevation. Previous studies have also reported that increased levels of these inflammatory mediators are indicative of impaired brain function (Grebenciucova and VanHaerents, 2023). Differences in the expression and activity of natriuretic peptide receptors between the brain and the heart may explain the limited effect of VS on IL-1β and IL-6 in brain tissue. Although the drug primarily exerts its anti-inflammatory effects by enhancing natriuretic peptide signaling, receptor density and downstream signaling are less pronounced in the brain, which may account for its reduced efficacy in this region (Ajay et al., 2023).

Furthermore, NF-kB pathway activation was observed in both the DOX and DOX + VS groups, associated with increased neuroinflammation. In addition, VS administration alone did not induce significant changes in NF-κB activity compared with controls, suggesting that the combination with DOX further activated the NF-κB pathway rather than inhibiting it, thereby potentiating the inflammatory cascade. These findings are consistent with previous reports showing DOX-induced NF-κB activation and neuroinflammation, whereas VS has been shown to attenuate NF-κB and inflammatory cytokines in cardiac tissue. The limited brain penetration of the active metabolite of sacubitril (LBQ657) provides a plausible explanation for the lack of neuroprotective effects. It may account for the novel observation that co-administration with DOX did not mitigate and may have failed to prevent NF-κB activation in the hippocampus (Alhowail and Aldubayan, 2023; Du et al., 2021; Ye et al., 2021).

While VS demonstrates significant cardioprotective properties, its effects within the central nervous system appear to diverge markedly. The neprilysin-inhibition component of VS (sacubitril) is particularly pertinent, given that neprilysin is a crucial brain endopeptidase responsible for the degradation of neuroactive peptides, such as amyloid-β, bradykinin, and substance P, which are known to activate microglia and stimulate the release of ROS, MDA, IL-1β, and TNF-α (Iwata et al., 2001; Nalivaeva et al., 2020; Skidgel and Erdös, 2004). Consequently, inhibiting neprilysin may facilitate the accumulation of these substrates in the brain, thereby creating a pro-inflammatory environment (Nalivaeva et al., 2020). Supporting this mechanism, several preclinical studies have indicated that neprilysin inhibition or sacubitril-containing regimens exacerbate amyloid-β deposition, microglial activation, and cognitive deficits compared to valsartan alone (Ali et al., 2023; Hersh and Rodgers, 2008; Zhang et al., 2024). Consistent with this evidence, our study revealed that VS alone significantly increased ROS, MDA, IL-1β, and TNF-α levels compared to the control, suggesting that VS can induce a low-grade oxidative stress and neuroinflammatory response under the current experimental conditions. Accordingly, the further elevation in IL-1β observed in the DOX + VS group is most plausibly attributed to the additive neuroinflammatory effects of DOX-induced cytokine activation, combined with the neprilysin inhibition-mediated accumulation of pro-inflammatory neuropeptides, rather than a failure of VS efficacy. Notably, there is no evidence that VS alters doxorubicin pharmacokinetics or systemic toxicity (Han et al., 2016; McMurray et al., 2014). Thus, the absence of neuroprotection and the enhanced cytokine response likely reflect brain-specific consequences of neprilysin inhibition and the limited capacity of VS or its active metabolite LBQ657 to traverse the BBB (Jung et al., 2025).

Our findings further elucidate that, in the context of apoptotic signaling, VS does not protect the hippocampus against DOX-induced cell death. Notably, caspase-3 activity was markedly increased in the DOX + VS group compared to DOX alone, suggesting an additive or synergistic pro-apoptotic effect when both treatments are combined. DOX is recognized for inducing intrinsic (mitochondria-dependent) apoptosis through mechanisms including excessive ROS production, mitochondrial DNA damage, cytochrome c release, and subsequent activation of caspase-9 and caspase-3 (Aldubayan et al., 2024). In our model, VS alone elevated ROS and pro-inflammatory cytokines, indicating that it may sensitize hippocampal neurons to apoptotic signaling rather than offering protection. Furthermore, the inhibition of neprilysin by VS could lead to the accumulation of neuroactive peptides, potentially promoting microglial activation, mitochondrial dysfunction, and caspase-3 activation (Nalivaeva et al., 2020; Iwata et al., 2001). In contrast to caspase-3, BAX expression in the DOX + VS group did not differ significantly from that in the DOX-alone group, suggesting that VS did not further affect the upstream BAX-mediated apoptotic initiation step. Collectively, these results suggest that VS enhances downstream apoptotic execution via caspase-3 activation while failing to mitigate DOX-induced mitochondrial apoptotic pathways.

Overall, these results highlight a distinct mechanistic difference between the cardiac and brain effects of VS. In the heart, neprilysin inhibition enhances natriuretic peptide signaling, increasing nitric oxide bioavailability and suppressing fibrosis, oxidative stress, and cytokine release. In the brain, neprilysin inhibition may reduce amyloid-β degradation and disrupt neuroprotective balance, potentially contributing to neuronal vulnerability (Garnier-Crussard, 2024). This mechanistic divergence explains why VS exhibits cardioprotection but fails to protect against DOX-induced neurotoxicity.

This study has several important strengths. A key strength is the consistent use of the same animal strain, age range, and sex, which minimizes biological variability. In addition, although numerous studies have documented the cardioprotective effects of VS, no prior studies have specifically evaluated its impact on Oxidative stress, neuroinflammation, apoptotic pathways, and cognitive function in DOX-treated rats. Notably, this study combined behavioral assessment, biochemical analysis, and histological evaluation of the hippocampus, providing a comprehensive understanding of both functional and structural changes in the brain. However, this study had some limitations. The absence of a valsartan-only treatment limits the ability to determine whether the lack of neuroprotective effect was driven by neprilysin inhibition, angiotensin receptor blockade, or a possible pharmacodynamic interaction between the two. The current study was designed primarily to assess the integrated effect of sacubitril/valsartan as a fixed-dose combination reflecting its clinical use. Nevertheless, future investigation incorporating a valsartan-only treatment group would provide valuable mechanistic insights. VS failed to confer neuroprotection; these findings highlight critical tissue-specific limitations of neprilysin inhibition and inform the design of future neuroprotective therapies.

In conclusion, this study demonstrated that VS was ineffective in protecting against DOX-induced cognitive impairment and hippocampal neurotoxicity in rats. These findings emphasize that the neuroprotective potential of neprilysin inhibition differs from its established cardioprotective actions. Further research is needed to investigate the requirements using more targeted molecular and cellular assays.

## Data Availability

The original contributions presented in the study are included in the article/supplementary material, further inquiries can be directed to the corresponding author.
